# Supplementary material for: Collaborative intelligence in AI: Evaluating the performance of a council of AIs on the USMLE
Source: PLOS Digit Health. 2025 Oct 9;4(10):e0000787. doi: 10.1371/journal.pdig.0000787 (PMC12510544; doi:10.1371/journal.pdig.0000787)
Supplement: S2 File — (ZIP) [file pdig.0000787.s002.zip › S2 Data Raw data files containing unprocessed question inputs and LLM outputs including transcripts of deliberations/STEP3_Question-110-1_Type-MC-NJ.docx]

**Facilitator** **[Round 1]:** A 7-year-old boy with sickle cell disease is brought to the office by his father because of fever and bone pain. The father says that his son was discharged 5 days ago after a 3-day hospitalization for the same symptoms. The patient had been admitted with a diagnosis of vaso-occlusive crisis with bone infarct, with resolution of symptoms after treatment with intravenous fluids, narcotics, and antibiotic therapy for 3 days. Blood cultures were negative. The patient's back pain and fever recurred 2 days ago and seem more severe than prior to the hospitalization. His medical chart shows that he had one other uncomplicated hospital admission at age 4 years for a vaso-occlusive crisis with lower extremity bone pain. Vital signs today are temperature 39.2°C (102.5°F), pulse 110/min, respirations 24/min, and blood pressure 115/60 mm Hg. The patient is uncomfortable but does not appear toxic. Cardiac examination discloses a grade 2/6 systolic ejection murmur at the left lower sternal border. Spleen is not palpated. There is considerable tenderness over the L1–L3 region of the back without fluctuation. There is diffuse pain in both thighs without localization, but range of motion is normal. Which of the following studies is most likely to establish the diagnosis at this time? (A) Blood culture (B) CT scan of the spine (C) MRI of the lumbar spine (D) Ultrasonography of the spine (E) X-ray of the spine

**AI_E:** The question presents a case of a 7-year-old boy suffering from sickle cell disease, an inherited disorder that affects the hemoglobin, and tends to cause misshapen red blood cells that can block arteries and cause painful vaso-occlusive crises. He previously had crises accompanied by fever and bone pain, resolved with hospitalization and various treatments. Recently, however, the symptoms returned more severe than before. Sickle cell disease causes numerous complications including acute bone crises, which can involve infarction and infection due to impaired circulation. The patient's back pain suggests an issue in the lumbar spine and the options given refer to medical investigations which can help in diagnosing the current crisis. Assuming that the patient is experiencing another vaso-occlusive crisis is one logical step given his medical history. However, several alternate assumptions can be considered, including infection (since he has fever), bone infarction (due to pain in the bone which can also result from sickle cell disease), and issues in the spinal region (as the child has severe pain over the L1-L3 region of the back). Prioritizing these assumptions clinically, we must consider that a severe vaso-occlusive crisis could have led to an issue within the spine, as the child has localized tenderness over the L1-L3 region. Sickle cell disease can result in bone infarcts due to vaso-occlusion, and in the spine, it could indicate vertebral osteomyelitis or avascular necrosis (bone death) caused by sickling and stasis in the microvasculature. In this case scenario, the most suitable diagnostic tool to visualize the marrow of the lumbar vertebrae for possible abnormalities would be an MRI of the lumbar spine. MRI is superior to a CT scan, ultrasonography or X-ray for evaluation of osteomyelitis, avascular necrosis, or bone marrow edema since it offers a detailed view of the soft tissues including the spinal cord, nerves, disks, and the vertebral bones. Blood culture, though important in assessing systemic infection, was previously negative, and given the specific pain in the lumbar region, an imaging study of the spine focused on visualizing the bone marrow appears more suitable. Finally, in direct response to the posed query, the most likely study that would establish this diagnosis at this stage is (C) MRI of the lumbar spine. This approach would allow for comprehensive visualization and pinpoint any abnormalities contributing to his symptoms.

**AI_B:** 1) RESTATING THE QUESTION: The query is about a 7-year-old young male with sickle cell disease, previously admitted for vaso-occlusive crises and recently discharged from the hospital, who is experiencing a recurrence of fever and bone pain more severe than before. A Vaso-occlusive crisis in patients with sickle cell disease refers to the blockage of blood circulation to the peripheral tissues due to the stickiness of their misshapen red blood cells, causing tissue and bone infarcts leading to extreme pain. The query is asking for the most appropriate diagnostic test to confirm the ongoing pathology at this time. 2) PLANNING: The immediate assumptions based on the information given are that he is having another vaso-occlusive crisis or that there may be an infection due to his immunocompromised state (caused by sickle cell disease). However, the severity of his symptoms coupled with the recent negative blood cultures could question the infection assumption. A further assumption could also be that he may have osteomyelitis or septic arthritis given his chronic sickle cell disease, associated bone infarcts, and recent bacterial infection. 3) CLINICAL REASONING: If the patient was experiencing a vaso-occlusive crisis, severe bone pain is expected, and it should have been controlled or resolved to some degree with the treatment given during his most recent hospitalization. The increased severity of the pain might suggest something else is going on—like osteomyelitis or septic arthritis. A vaso-occlusive crisis also wouldn't explain the fever whereas infection rapid onsets would, either bacterial or from other sources. Given the localized tenderness over the lumbar region, the assumption leans towards bone pathology—specifically osteomyelitis, which could have been resultant of past bone infarcts and recent bacterial infection. 4) CONCLUDING: An examination that allows visualization of the bony structures, particularly the lumbar area considering where the pain localized, would be most helpful in providing a differential diagnosis. With the above assumptions in mind, an imaging study like an MRI of the lumbar spine (Option C) would be most likely to reveal bone abnormalities or signs of osteomyelitis. 5) RESPONSE: Therefore, the most suitable study to establish the diagnosis at present is likely to be an MRI of the lumbar spine (Option C). If osteomyelitis is found, it may explain the severe pain and recurrent fevers, and appropriate antibiotic therapy can then be initiated based on culture and sensitivity test results.

**AI_D:** RESTATING THE QUESTION: So, we are presented with a 7-year-old boy with sickle cell disease who has a history of recurrent hospital admissions due to what is thought to be a vaso-occlusive crisis. He has come back with the same symptoms of fever and bone pain, which are now seemingly more severe than before. His vital signs show an elevated temperature indicative of a potential infection or inflammation. His physical exam didn't exhibit significant abnormalities apart from a systolic murmur and tenderness in the lower back region. The spleen was not enlarged. We are asked to decide what diagnostic test would be most beneficial in determining his current illness. PLANNING: Key factors here are recurrent bone pain in a sickle cell disease patient, worsening of symptoms with time, and inconclusive results of prior blood cultures. His sickle cell disease puts him at risk for conditions like vaso-occlusive crisis, bone infarctions, but also the risk of infection from encapsulated organisms and avascular necrosis. This leads me to hypothesize that his current condition also might be due to an issue localized in the bone like osteomyelitis, vertebral disc infection or avascular necrosis instead of systemic infection. CLINICAL REASONING: His physical exam shows tenderness when pressure is exerted on his lower back area, but his spleen is normal, and his cardiac exam only reveals a minor systolic murmur which can be a common finding in children. Blood cultures have proven to be negative. This seems to point away from systemic infection and towards bone-related issues. Bone infections like osteomyelitis can be hard to culture. The pain in his back and thighs can also be related to avascular necrosis, a condition caused by insufficient blood supply leading to bone cell death, which can be rampant in sickle cell disease due to vaso-occlusion. CONCLUDING: During a vaso-occlusive crisis, the blood vessels get blocked by sickled red blood cells inhibiting blood flow to the tissues, and the bone tissue is especially susceptible to this. Hence it seems feasible that the boy might be suffering from a condition related to bone, like vertebral disc infection, osteomyelitis, or avascular necrosis. RESPONSE: Thus, to visualize soft tissues like bone marrow or spinal discs in detail, MRI is the ideal diagnostic tool. A CT scan can also provide good details but it won't match up to the sensitivity and specificity of an MRI in this case. Thus, the answer would be (C) MRI of the lumbar spine.

**AI_C:** 1) RESTATING THE QUESTION: The question presents a 7-year-old boy with a history of sickle cell disease who is exhibiting recurrent fever, back pain, and bone pain. These symptoms have led to multiple hospitalizations for what has been diagnosed as a vaso-occlusive crisis and bone infarct. The current bout of symptoms is more severe than previous occasions. The physical examination reveals a grade 2/6 systolic murmur on cardiac auscultation, and the patient shows tenderness in the L1-L3 region of the back but without any local fluctuation. There is also unlocalized pain in both thighs. Despite the pain, the patient's range of motion remains normal. The question is asking us what diagnostic test would be most appropriate to establish a diagnosis based on the current symptoms. 2) PLANNING: To proceed with the clinical reasoning process, we first need to clarify our assumptions. The boy's past medical history and physical examination point towards sickle cell disease complications, more specifically, perhaps another vaso-occlusive crisis. However, the worsening of symptoms and lack of complete resolution despite standard treatment for vaso-occlusive crisis challenge this assumption. Therefore, an alternate assumption could be that the boy might be suffering from another complication of sickle cell disease other than a simple vaso-occlusive crisis. Despite the recurrence of symptoms similar to a vaso-occlusive crisis, the patient's current condition could be due to an osteomyelitis, which is common in patients with sickle cell disease. 3) CLINICAL REASONING: In patients with sickle cell disease, the infarcts caused during vaso-occlusive crises provide a fertile ground for bacterial infections which lead to osteomyelitis. The intact range of motion despite pain, the increased pain severity, and the recurrent nature of pain even after resolution post-treatment suggest the presence of a disease process in addition to vaso-occlusion. Considering the mentioned assumptions, differentiating between vaso-occlusive crisis and osteomyelitis becomes crucial because the management plan would differ depending upon the diagnosis. 4) CONCLUDING: Keeping in mind the clinical presentation of the patient, the recurrence of symptoms post-treatment, and our alternate assumption that the patient might be affected by osteomyelitis, a diagnostic modality that can offer detailed images of bone structure and highlight possible areas of infection would be preferred. Such a diagnostic tool is the MRI, which is superior to the other options in identifying the early stages of osteomyelitis, as well as changes in bone marrow consistent with vaso-occlusive crises, thus offering a high-resolution contrast between normal and pathological tissues. 5) RESPONSE: Therefore, the most appropriate investigative study to establish the diagnosis at this time would be (C) MRI of the lumbar spine.

**AI_A:** (1) RESTATING THE QUESTION: The boy, who has sickle cell disease, is experiencing fever and bone pain. He was recently hospitalized and treated for a vaso-occlusive crisis with bone infarcts, a medical condition in which sickled cells block blood vessels, leading to tissue death due to lack of oxygen. Although his symptoms improved with treatment, they have recurred and seem worse than they were before. His medical history includes another vaso-occlusive crisis when he was 4. On examination, he is uncomfortable, has a systolic ejection murmur and tenderness over the lumbar region of the back, but no splenic enlargement or detection of abscesses (fluctuation). The question asks what diagnostic study is most likely to help establish the diagnosis at this point. (2) PLANNING: The primary assumption from the history and physical examination is that the patient is having another severe vaso-occlusive crisis, potentially involving his long bones and back. The recurrent symptoms might suggest a progression of his disease, leading to complications such as bacteremia or osteomyelitis. However, we can question this assumption by asking: is this really another typical vaso-occlusive crisis or could it be a different complication of sickle cell disease like avascular necrosis, osteomyelitis or a vertebrae infarct? Evaluating these, we hypothesize that given the severity and localization of his symptoms, a complication involving his bones, particularly osteomyelitis or vertebrae infarct, should be clinically prioritized to pursue. (3) CLINICAL REASONING: Children with sickle cell disease can frequently present with vaso-occlusive crises, usually characterized by bone pain due to bone marrow ischemia or infarcts. However, the persistence and worsening of this patient's symptoms despite treatment and hospitalization suggest a more severe problem. Given the tenderness over the L1–L3 region of the spine, it indicates involvement of the vertebrae. Osteomyelitis caused by Salmonella or Staphylococcus aureus is commonly seen in patients with sickle cell disease, but the lack of fluctuation makes abscess less likely. Additionally, the patient’s blood culture came back negative previously. Bone infarction, particularly in vertebral bodies, can also happen as a consequence of vaso-occlusive crisis, presenting with severe pain over the affected area. (4) CONCLUDING: The examination findings and the history of the patient direct us towards a condition that would cause severe localized pain, such as osteomyelitis or vertebrae infarct. Still, the absence of findings like fluctuation makes osteomyelitis less likely. Given this, a vertebral body infarction seems a more plausible explanation for this patient's symptoms and should therefore be investigated. (5) RESPONSE: Considering all these factors, the best study to establish the diagnosis would be one that allows visualization of the lumbar spine, specifically soft tissues, bone marrow and trabecular bone. Therefore, the answer should be (C) MRI of the lumbar spine.

**Facilitator** The consensus among the AI responses is that the most appropriate diagnostic study for the 7-year-old boy with sickle cell disease, who is experiencing recurrent fever and bone pain, would be an MRI of the lumbar spine (Option C). This conclusion is based on the boy's medical history, his recent hospitalization for a vaso-occlusive crisis with bone infarct, and the recurrence of symptoms that seem more severe than before. The boy's physical examination reveals tenderness over the L1–L3 region of the back, suggesting a potential issue within the spine. While the initial assumption might be that the boy is experiencing another vaso-occlusive crisis, the worsening of symptoms and lack of complete resolution despite standard treatment challenge this assumption. Therefore, an alternate hypothesis is that the boy might be suffering from another complication of sickle cell disease, such as osteomyelitis or vertebral body infarction. Given the severity and localization of his symptoms, a diagnostic modality that can offer detailed images of bone structure and highlight possible areas of infection would be preferred. An MRI of the lumbar spine is superior to other options in identifying the early stages of osteomyelitis, as well as changes in bone marrow consistent with vaso-occlusive crises, thus offering a high-resolution contrast between normal and pathological tissues. Therefore, the consensus is that the most appropriate investigative study to establish the diagnosis at this time would be an MRI of the lumbar spine.
